# Influence of Contrast Agent Injection Scheme Customized by Dual-Source CT Based on Automatic Tube Voltage Technology on Image Quality and Radiation Dose of Coronary Artery Imaging

**DOI:** 10.3389/fsurg.2022.862697

**Published:** 2022-04-05

**Authors:** Weiling He, Xin Chen, Rui Hu, Wenjie Sun, Weili Tan

**Affiliations:** ^1^Department of Radiology, Hunan Provincial People's Hospital, The First Affiliated Hospital of Hunan Normal University, Changsha, China; ^2^Diagnostic Radiology Department, Hunan Cancer Hospital, Changsha, China; ^3^Interventional Vascular Surgery Department, Hunan Provincial People's Hospital, The First Affiliated Hospital of Hunan Normal University, Changsha, China

**Keywords:** coronary artery, coronary CT angiography, image quality, radiation dosage, aortic root

## Abstract

**Objective:**

To explore the influence of a contrast agent injection scheme customized by dual-source CT based on automatic tube voltage technology on coronary imaging image quality and radiation dose.

**Methods:**

A total of 205 patients who underwent coronary CT angiography (CCTA) in our hospital from June 2021 to September 2021 were selected. 105 patients in the control group who underwent routine scanning according to body mass (BMI) and 100 patients in the observation group who set tube voltage and contrast agent dosage according to automatic tube voltage selection technology. CT values of the aortic root (AO); left anterior descending (LAD) branch; proximal, middle, and distal segments of the right coronary artery (RCA); and proximal and distal segments of left circumflex (LCX) branch were measured. We calculated the signal-to-noise ratio (SNR) and contrast-to-noise ratio (CNR) of the image. Image quality scoring and effective dose (ED) calculation were carried out.

**Results:**

There was no significant difference in the CT value, SNR value, and CNR value of each part of the artery between the two groups (*P* > 0.05). Image quality scores of the control group and the observation group were 1.28 ± 0.25 and 1.25 ± 0.23, respectively, and there was no significant difference in scores (*P* > 0.05). In the control group, the dosage of comparator was 43.81 ± 6.74 ml, and the ED was 4.92 ± 1.26 mSv. The dosage of contrast agent in the observation group was 34.23 ± 6.39 ml, and ED was 3.05 ± 0.94 mSv. The dosage of contrast agent and ED in the observation group were lower than those in the control group (*P* < 0.05).

**Conclusion:**

The contrast agent injection scheme customized by dual-source CT based on automatic tube voltage technology can meet the clinical requirements of coronary image quality, reduce the radiation dose and contrast agent consumption, and help doctors choose a more accurate and reasonable examination scheme, which has certain clinical application value.

## Introduction

With the acceleration of population aging, the prevalence trend of cardiovascular risk factors is obvious in China, which leads to increase in the number of cardiovascular diseases. Coronary CT angiography (CCTA) has become an important method for clinical diagnosis and investigation of coronary heart disease because of its fast imaging speed and few complications (1). In the past, traditional CT was limited by detector width, and respiratory artifacts caused more interlaced images, which restricted the examination. However, CCTA can collect accurate and safe clinical information of human tissues and organs in the physiological state during scanning, and its application has gained rapid popularity (2). However, the high radiation dose related to CCTA has become one of the key problems that limit its further promotion and application in routine screening. In past clinical CCTA scanning, to ensure image quality, scanning tube voltage and contrast agent injection dose were usually selected according to the patient's body mass index, which lacked individualized and standardized specifications and needed further research and discussion (3).

Foreign studies have shown that automatic tube voltage selection facilitates CCTA image acquisition, and that it is feasible to customize the contrast injection protocol based on automatically selected tube voltage level (4). CT radiation dose is closely related to scanning parameters. In recent years, research on the relationship between tube current and radiation dose have mostly focused on the automatic tube current setting technology, which is a technology for automatically adjusting tube current according to a scout image and can obviously reduce radiation dose in the abdominal and pelvic scanning. Based on the automatic tube voltage technology, this study adjusts the dosage of a contrast agent to explore the feasibility of CCTA image acquisition and its influence on radiation dose to provide reference for clinical CCTA examination.

## Data and Methods

### General Information

A total of 205 patients who underwent CCTA in our hospital from June 2021 to September 2021 were selected. Inclusion criteria were patients with suspected coronary heart disease, no history of iodine contrast agent allergy, no history of coronary intervention or coronary artery bypass surgery, good breath-holding training, and nonpregnant or lactating women. Exclusion criteria were severe arrhythmia, cardiac insufficiency, severe hepatic and renal insufficiency, implantation of a pacemaker, and coronary artery bypass grafting. According to different contrast agent injection schemes, 105 patients in the control group were selected tube voltage and contrast agent dosage based on patient body mass, and 100 patients in the observation group were set tube voltage and contrast agent dosage by automatic tube voltage selection technology. There were 107 men and 98 women, with age of 59.26 ± 9.16 years and BMI of 25.74 ± 3.09 kg/m^2^. This study was approved by the hospital ethics committee and informed consent of the patients and their families.

### Research Methods

All examinations were performed by 3rd generation dual-source CT (SOMATOM Definition Force) and prospective ECG gating sequence scanning. The patients took the supine position, and CCTA was performed from the thoracic inlet to the cardiac diaphragmatic surface, with the scanning range ranging from 1 cm below the tracheal bifurcation to the cardiac diaphragmatic surface level. Before the examination, all the patients were trained to hold their breath, including taking nitroglycerin 0.5 mg. A contrast agent (Iohexol injection 370 mg/ml) was injected first, and then physiological saline was injected at the same flow rate; all of which were injected with a double-cylinder high-pressure syringe. When the proximal level of the ascending aorta reaches the trigger threshold of 100 HU, automatic scanning will be delayed for 5 s. Scanning parameter settings were: acquisition layer thickness 0.75 mm, layer spacing 0.4 mm, and rotation time 0.25 s.

For the control group, the dosage of contrast agent was set according to body mass (< 50 kg, 32 ml; 50–59 kg, 36 ml; 60–69 kg, 40 ml; 70–79 kg, 45 ml; ≥ 80 kg, 50 ml), and injection time was 12 s. Patients in the observation group used the automatic tube voltage selection technology to set the dosage of contrast agent (70 kV, 33 ml; 80 kV, 36 ml; 90 kV, 42 ml; 100 kV, 46 ml; 110 kV, 52 ml; 120 kV, 55 ml), and injection time was 10 s (5). All the patients' images were transmitted to the image post-processing workstation (Siemens Healthcare, Forchheim, Germany), and multi-planar reformation (MPR), volume rendering (VR), and curved planar reformation (CPR) were performed for image reconstruction and post-processing.

The CT values of the proximal, middle and distal segments of the aortic root (AO), left anterior descending branch (LAD), right coronary artery (RCA), and left circumflex branch (LCX) were measured. The standard deviation of the mean CT value of the AO region of interest (ROI) was taken as the image noise. The ROI should be as large as possible, and we avoided areas, such as coronary vessel wall, calcified plaque, and non-calcified plaque, as much as possible, where the ROI of the aortic root was about 200 mm^2^, to ensure the accuracy of the measured CT values. Calculated signal-to-noise ratio (SNR) and contrast-to-noise ratio (CNR), SNR = CT value of AO/image noise of AO. CNR = (target blood vessel CT value-fat CT value)/AO image noise.

The coronary artery tree was divided into 18 segments using the newly recommended 18-segment standard modified segmentation method by the American Cardiovascular CT Association, and the distal segment of the occluded vessel was excluded from the analysis (6). Image quality was scored by two senior radiologists through the improved segmentation method of 18-segment standard, with the scoring standard of 1–4 points:1 point: excellent blood vessel display, no ladder-like artifact; 2 points: slight pulsation artifact in the blood vessel; 3 points: moderate artifact in the blood vessel; 4 points: unclear blood vessel display or severe ladder-like artifact that cannot be evaluated. In case of disagreement, an agreement was reached through consultation. We recorded the product of dose length and calculated the effective dose (ED) (7) [ED = dose length product × conversion coefficient k (k = 0.014 mSv·mGy^−1^·cm^−1^)].

For the repeatability test, images of 15 patients were randomly selected from the control group and the observation group. Two physicians performed image quality analysis separately. The Kappa value was used to evaluate the consistency of image quality between the two physicians (*kappa* > 0.8). One week later, another image quality analysis was performed by one of the physicians to calculate intra-examiner consistency (Cronbach's α = 0.927).

### Statistical Methods

The SPSS 22.0 software was used to process experimental data. Measurement data, such as age, BMI, CT value, SNR, CNR, and ED, of the patients were expressed as mean plus standard deviation ($\bar{x}$ ± s), and the *t*-test was performed for pairwise comparison. Enumeration data, such as gender, of the patients were expressed, in %, and comparison was conducted by the χ^2^ test. Test level was α = 0.05, and difference was statistically significant when *P* < 0.05.

## Results

### Comparison of General Data of the Two Groups of Patients

There was no significant differences in general information such as age, gender, BMI and heart rate between the observation group and the control group (*P* > 0.05). As shown in Table 1.

**Table 1:** Comparison of general information between the two groups of patients (*n*, $\bar{x}$ ± s).

**Group**	**Age** **(years)**	**Gender (male/ female)**	**Body mass index (kg/m^**2**^)**	**Heart rate (beats/min)**
Control group (*n* = 105)	58.41 ± 8.36	54/51	25.63 ± 2.41	62.19 ± 7.48
Observation group (*n* = 100)	60.15 ± 10.07	53/47	25.86 ± 2.59	63.05 ± 7.82
*t*/*χ^2^ value*	1.349	0.051	0.659	0.805
*P-value*	0.179	0.822	0.511	0.421

### CT Comparison of AO and Coronary Artery Segments Between the Two Groups

There is no significant difference in CT values of AO, LAD, RCA, and LCX between the two groups (*P* > 0.05), as shown in Table 2.

**Table 2:** Comparison of CT of coronary artery segments between the two groups (*n*, $\bar{x}$ ± s, HU).

**Group**	**AO**	**LAD-p**	**LAD-m**	**LAD-d**	**RCA-p**	**RCA-m**	**RCA-d**	**LCX-p**	**LCX-d**
Control group (*n* = 105)	491.72 ± 85.13	483.16 ± 68.49	449.71 ± 63.45	412.87 ± 56.27	493.58 ± 81.06	462.71 ± 74.25	446.27 ± 65.37	472.64 ± 78.92	413.27 ± 51.74
Observation group (*n* = 100)	498.26 ± 89.53	470.81 ± 61.28	437.87 ± 59.38	401.52 ± 55.03	486.92 ± 73.54	458.49 ± 69.81	439.61 ± 60.28	465.26 ± 71.23	402.78 ± 50.29
*t value*	0.454	1.358	1.378	1.459	0.615	0.418	0.757	0.702	1.471
*P-value*	0.650	0.176	0.170	0.146	0.539	0.675	0.450	0.484	0.143

*LAD-p, proximal to the left anterior descending coronary artery; LAD-m, middle segment of the left anterior descending coronary artery; LAD-d, distal left anterior descending coronary artery; RCA-p, proximal right coronary artery; RCA-m, middle right coronary artery; RCA-d, distal right coronary artery; LCX-p, proximal left circumflex branch; LCX-d, distal left circumflex branch*.

### Comparison of SNR and CNR Between the Two Groups of Patients

There was no significant difference between the two groups in SNR and CNR of LAD, RCA and LCX (*P* > 0.05), as shown in Table 3.

**Table 3:** Comparison of signal-to-noise ratio (SNR) and contrast-to-noise ratio (CNR) between the two groups of patients (*n*, $\bar{x}$ ± s, HU).

**Group**	**SNR**	**CNR**							
		**LAD-p**	**LAD-m**	**LAD-d**	**RCA-p**	**RCA-m**	**RCA-d**	**LCX-p**	**LCX-d**
Control group (n = 105)	28.16 ± 6.24	24.71 ± 6.39	22.74 ± 5.92	21.43 ± 5.58	24.27 ± 6.71	23.04 ± 5.26	23.81 ± 6.33	24.17 ± 6.92	21.93 ± 4.87
Observation group (n = 100)	27.53 ± 5.71	23.19 ± 5.18	21.57 ± 4.86	20.97 ± 4.63	23.69 ± 5.78	22.47 ± 5.09	22.75 ± 5.12	22.86 ± 5.35	20.57 ± 4.42
*t value*	0.753	1.865	1.542	0.641	0.662	0.788	1.314	1.511	0.553
*P value*	0.452	0.064	0.125	0.523	0.509	0.431	0.190	0.132	0.581

*LAD-p, proximal to the left anterior descending coronary artery; LAD-m, middle segment of the left anterior descending coronary artery; LAD-d, distal left anterior descending coronary artery; RCA-p, proximal right coronary artery; RCA-m, middle right coronary artery; RCA-d, distal right coronary artery; LCX-p, proximal left circumflex branch; LCX-d, distal left circumflex branch*.

### Comparison of Image Quality Between the Two Groups of Patients

**Figure 1:**
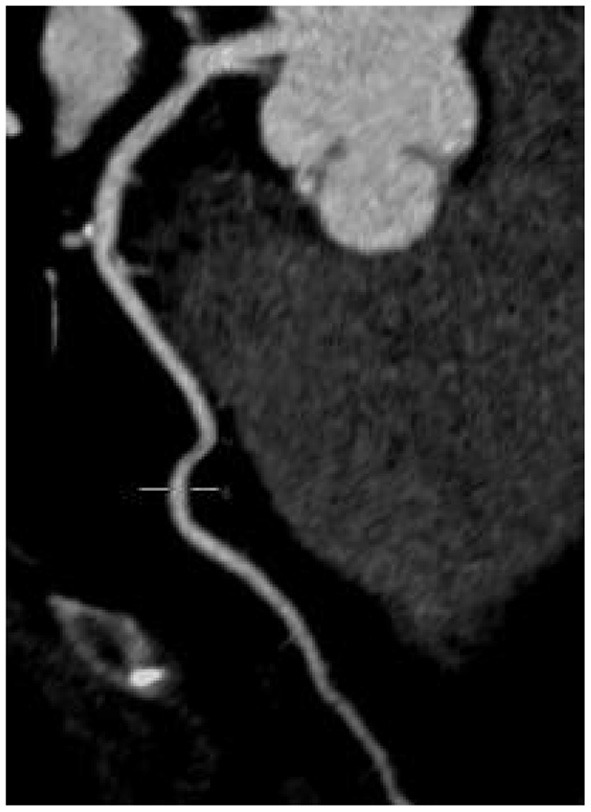
Typical image of left anterior descending curved planar reconstruction in the control group.

**Figure 2:**
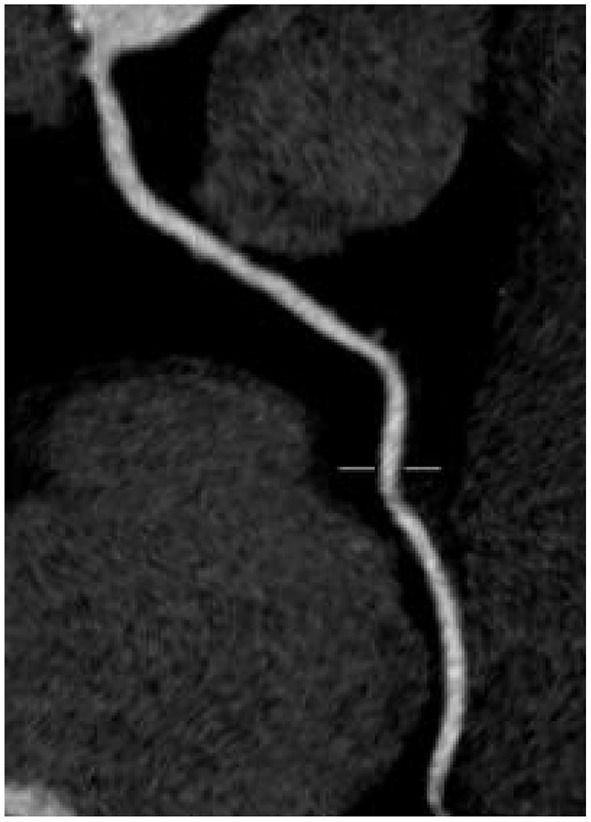
Typical image of right coronary artery curved planar reconstruction in the control group.

**Figure 3:**
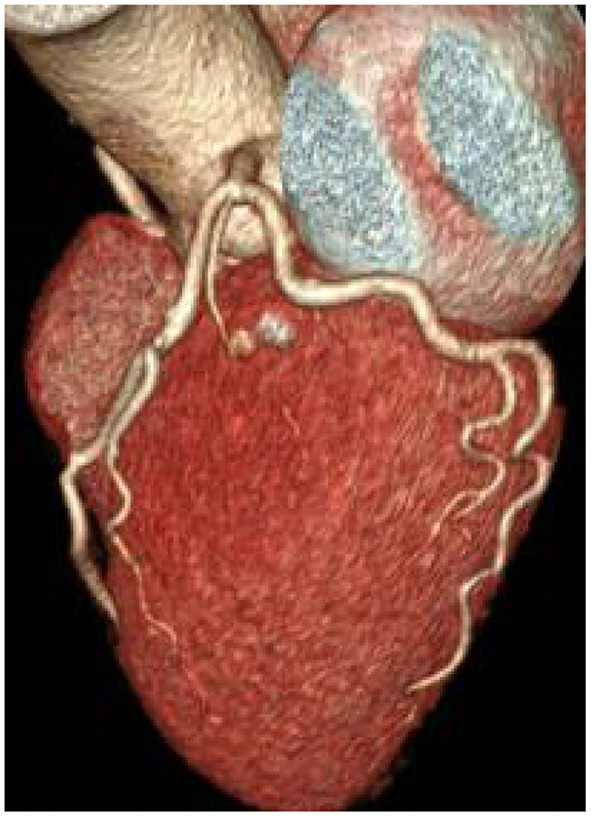
Typical image of coronary CT angiography (CCTA) volume rendering in the control group.

**Figure 4:**
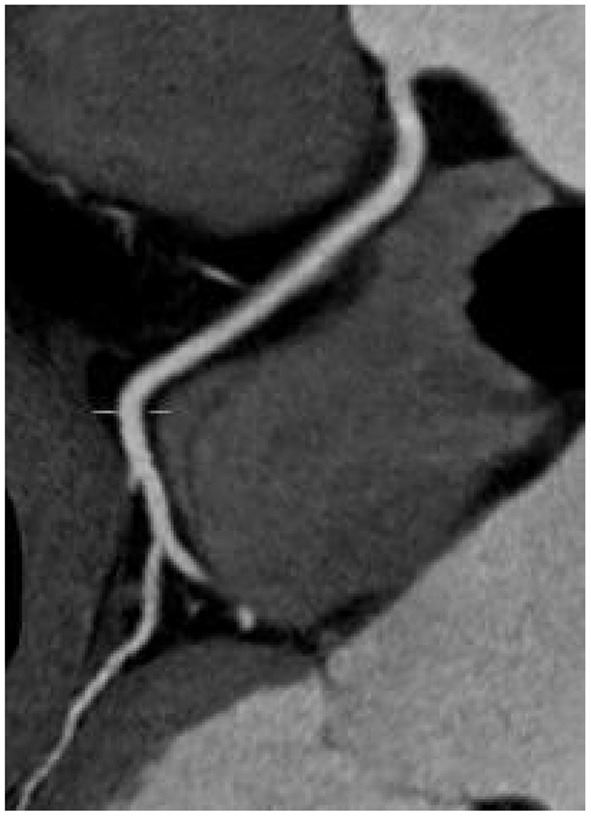
Typical image of left anterior descending curved planar reconstruction in the observation group.

**Figure 5:**
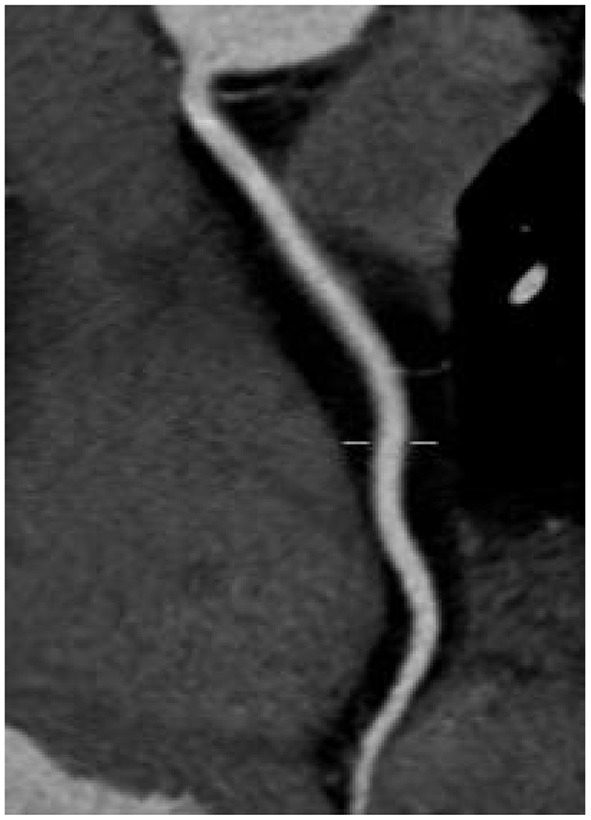
Typical image of right coronary artery curved planar reconstruction in the observation group.

**Figure 6:**
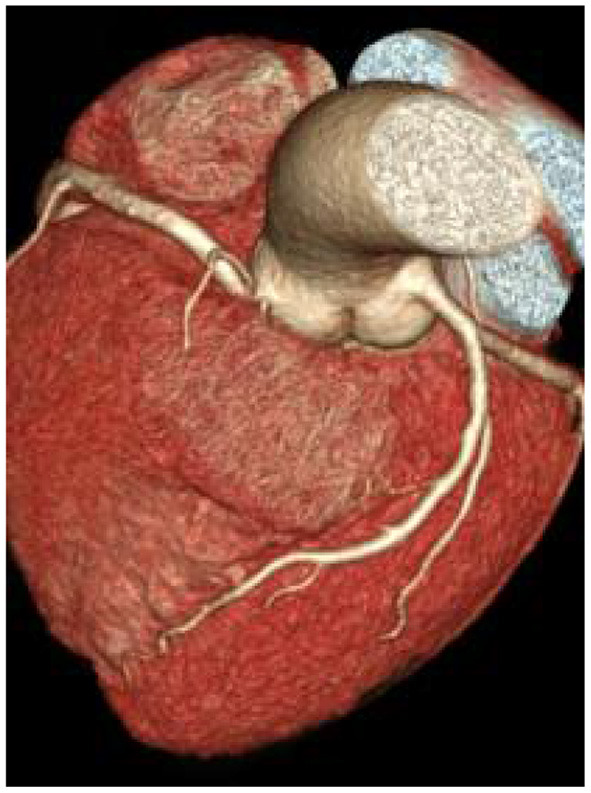
Typical image of CCTA volume rendering in the observation group.

There was no significant difference in image quality scores between the two groups (*P* > 0.05), as shown in Figures 1–6. Figures 1–3: a patient in the control group, male, 55 years old, BMI 25.2 kg/m^2^, tube voltage 80 kv and contrast agent dosage 42 ml during scanning; image quality score is 1 point, average CT value of AO is about 603 HU, noise value is 26 HU, and ED is 2.7 mSv. Figures 4–6: a patient in the observation group, female, 61 years old, BMI 25.8 kg/m^2^, tube voltage 80 kv, and contrast agent dosage 35 ml during scanning; image quality score is 2 points, average CT value of AO is 533 HU, noise value is 25 HU, and ED is 1.9 mSv.

### Comparison of Contrast Agent Dosage and ED Between the Two Groups of Patients

**Figure 7:**
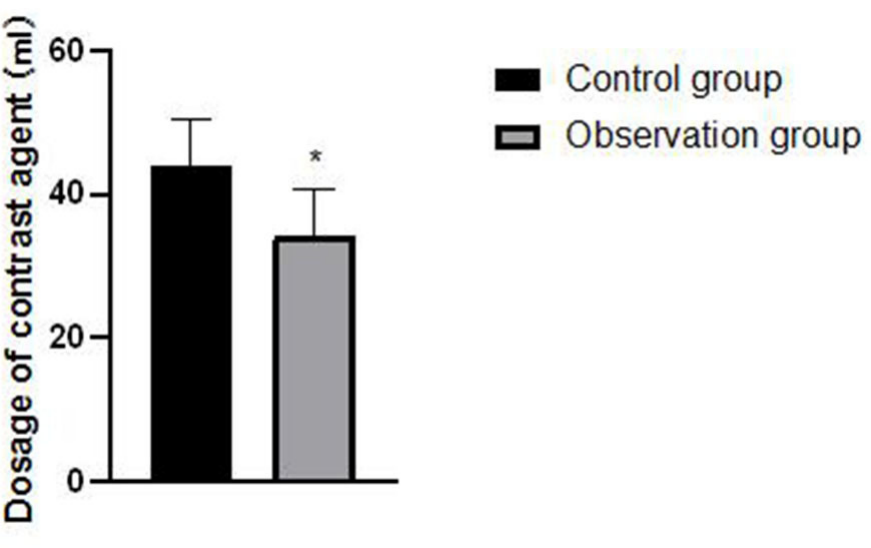
Comparison of contrast agent dosage between the two groups of patients. Compared with the control group, *P < 0.05.

**Figure 8:**
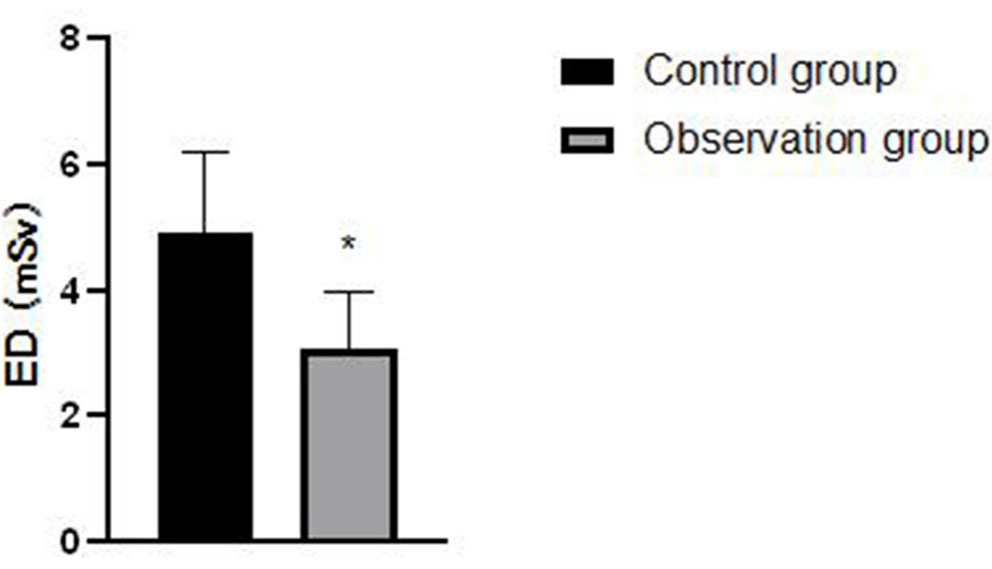
Comparison of effective dose (ED) between the two groups of patients. Compared with the control group, *P < 0.05.

The dosage of contrast agent and ED in the observation group are lower than those in the control group, and the differences are statistically significant (*P* < 0.05), as shown in Figures 7, 8.

## Discussion

With the rapid development of multi-slice spiral CT technology, coronary CTA has become one of the first-choice technologies for screening coronary heart disease safely and reliably. However, the high radiation dose associated with coronary CTA examination and cancer risk, radiation protection, and safety problems caused by ionizing radiation have gradually become the focus of widespread concern in society. On the website of the US Food and Drug Administration, the statement of the American Heart Committee is published, which concludes that a CT radiation dose of 10 msv can cause a malignant tumor in 1/2,000 patients undergoing CT (8). Therefore, how to effectively reduce radiation dose while ensuring image quality has become a research hotspot.

At present, the most commonly used methods to reduce radiation dose include reducing tube voltage and current, reducing scanning length, forward-looking ECG trigger scanning, large pitch scanning, and new reconstruction algorithms. Radiation dose is proportional to the square of the tube voltage, which indicates that reducing tube voltage is the most effective way to reduce radiation dose at present (9, 10). Chen et al. studied head and neck CTA scanning with tube voltage reduced to 70 KV, and image quality score had no obvious change, but radiation dose could be reduced by about 58%, which reflected the advantages of automatic tube voltage technology in reducing radiation dose (11).

Based on the customized scheme of automatic tube voltage technology, the lowest or next lowest tube voltage can be selected for scanning according to inspection purpose, and parameters meeting image quality requirements can be set, which provides the basis for further reducing the contrast agent (12, 13). The results show that there is no significant difference in the AO, SNR, CT, and CNR values of each segment of the coronary artery between the two groups. It is suggested that the objective image quality of CCTA scanned with the automatic tube voltage technology is consistent with that of the selection of tube voltage and contrast agent dose according to a patient's body mass, and image quality has not been reduced. This is consistent with the research of Ippolito et al. (14).

In the past, CCTA examination was limited by actual situation, and tube voltage was selected empirically according to the body mass of patients. However, for patients with the same body mass range, some patients needed higher tube voltage levels to obtain better scanning images because of differences in body shape and muscle and adipose tissue content distribution, and some patients with larger body mass only needed conventional or even lower tube voltage to meet the examination requirements and obtain better image quality (15, 16). For patients with normal and same body mass range, using the 3rd generation Shuang Yuan CT automatic tube voltage selection technology, the tube voltage of CCTA examination can be intelligently selected, and effects of other patient parameters on the examination besides body mass should be considered, so as to help technicians to more accurately and reasonably select the optimal voltage value suitable for patients. It is necessary to improve the success rate of examination and ensure image quality while effectively controlling or reducing radiation dose (17, 18). In this study, there was no significant difference in image quality scores between the two groups, but the dosage of contrast agent and ED in the observation group were significantly lower than those in the control group. It is suggested that the customized scheme based on automatic tube voltage technology can take into account the influence of other patient parameters except body mass on examination, thus helping technicians to select the best voltage value suitable for patients more accurately and reasonably, and effectively controlling or reducing radiation dose while improving the success rate of examination and ensuring image quality.

Previous studies have shown that the automatic tube voltage technology can automatically adjust tube voltage and make appropriate compensations according to various factors, such as cardiac output, body shape, and chest shape of different patients. At the same time, according to the proportional relationship between radiation dose and tube voltage, the purpose of reducing radiation dose can be achieved by adjusting tube voltage (19). Most the iodine contrast agents used in CCTA examinations are hypertonic, and iodine content is as high as 37%. In the human body, iodine contrast agents are filtered by glomerulus but not absorbed by renal tubules. During the process of glomerular filtration, concentration in the kidney increases, which may lead to a series of adverse reactions, such as renal damage (20). Low contrast agent flow rate and low contrast agent dose usually reduce the enhancement density of coronary arteries. By reducing tube voltage and adjusting the dose of a contrast agent, injection flow rate can be kept at a level slightly lower than that of conventional injection flow rate (6 ml/s), Compton scattering effect can be reduced, and the dose of the contrast agent and radiation can be reduced while meeting image quality requirements.

There are some shortcomings in this study, including the small number of selected cases and lack of gold standard contrast in coronary angiography, which need to be supplemented and further discussed in follow-up research studies.

To sum up, the contrast agent injection scheme customized by dual-source CT based on the automatic tube voltage technology can meet the clinical requirements of coronary imaging image quality, reduce radiation dose and contrast agent consumption at the same time, and help doctors choose a more accurate and reasonable examination scheme, which has certain clinical application value.

## Data Availability Statement

The original contributions presented in the study are included in the article/supplementary material, further inquiries can be directed to the corresponding author.

## Ethics Statement

The studies involving human participants were reviewed and approved by the Hospital Ethics Committee of the Hunan Provincial People's Hospital. The patients/participants provided their written informed consent to participate in this study. Written informed consent was obtained from the individual(s) for the publication of any potentially identifiable images or data included in this article.

## Author Contributions

All authors listed have made a substantial, direct, and intellectual contribution to the work and approved it for publication.

## Funding

This work was funded by 2020 Scientific Research Project of Hunan Health Committee (No. 20200059).

## Conflict of Interest

The authors declare that the research was conducted in the absence of any commercial or financial relationships that could be construed as a potential conflict of interest. The reviewer XP declared a shared affiliation with the authors XC and WT to the handling editor at the time of review.

## Publisher's Note

All claims expressed in this article are solely those of the authors and do not necessarily represent those of their affiliated organizations, or those of the publisher, the editors and the reviewers. Any product that may be evaluated in this article, or claim that may be made by its manufacturer, is not guaranteed or endorsed by the publisher.
